# Supplementary material for: SyNDI: synchronous network data integration framework
Source: BMC Bioinformatics. 2018 Nov 6;19:403. doi: 10.1186/s12859-018-2426-5 (PMC6219086; doi:10.1186/s12859-018-2426-5)
Supplement: Supplementary file 5 — Files of the example presented in Additional file 4. This additional file is a zip package that contains all files used in Additional file 4. Detailed descriptions of each of these files are presented in a README file included in this zip package. (GZ 30411 kb) [file 12859_2018_2426_MOESM5_ESM.gz › SyNDITools/Meme2Fimo/meme_4.9.0_4.tar.gz!/meme_4.9.0/etc/centrimo_template.html]

Centrimo


Each "match-probability curve" shows the probability of the
**best** match to a given motif occuring at a given position in the
input sequences. This probability is based only on sequences that
contain at least one match with score greater than the minimum score defined
for this motif.

Points (X,Y) on the plot are:  
  Y = Pr(best match occurs at position X | sequence contains a match)

**Note:** The plots are smoothed according to the function
selected from the "Smoothing" menu on the right. Setting the smoothing
window size to 1 turns off smoothing.

If a negative dataset has been supplied then two lines are drawn for
each motif representing both datasets. The normal dataset is plotted with
a single unbroken line whereas the negative dataset is plotted with a
dashed line.

[
close ]

This shows a listing of all motifs currently plotted on the graph.

The color used to plot a motif can be changed by clicking on the
color swatch next to the motif you want to change, followed by clicking
on the color swatch you wish to swap it with.

[
close ]

These are extra colors you may use for plotting motifs.

Click on the color swatch next to one of the above motifs, then click
on one of these "unused color" swatches to change the color of the
motif's plot.

[
close ]

These options change the display of the graph.

Smoothing:
:   Allows selection of the smoothing function applied to the graph.

    The weighted moving average option uses weights shaped as an isosceles
    triangle where the central point (or points in an even sized window)
    get the maximum weight.

    The moving average simply weights all points in the smoothing window
    equally.

    **Note:** Setting the smoothing window size to 1 turns off
    smoothing.

Window
:   The window size used to smooth the graph. The larger the smoothing
    window size, the smoother the graph, at the cost of hiding detail.

    Below a smoothing window size of 10, thinner lines are used on the
    graph to allow more detail to be visible.

    **Note:** Remember to press "return" or "enter" after changing
    the number in the input box in order to see the effect of the new
    smoothing window size.

Legend
:   Choose to display/disable the on-graph legend. The legend can be
    moved by clicking on the graph.

Negative Sequences
:   Choose whether to plot the motif probability curve(s) for the
    negative sequences (if provided). The curve(s) are plotted as dashed
    lines, using the same color as the corresponding curve for the positive
    sequences.

Download EPS
:   Download the graph that you are currently viewing as an
    encapsulated postscript (EPS) image. EPS images are scalable making them
    suitable for publication.

[
close ]

List only enriched motifs that meet the selected filter criteria below.

**Selected motifs are always listed**; deselect all motifs first by clicking on
the "X" above the color swatches if you wish to filter all motifs.

To filter on "ID" or "Name", you can enter any Javascript regular
expression pattern. See here
for documentation on Javascript regular expression patterns.

[
close ]

Sorting is applied after filtering where possible (the exception being
the "Top" filter) so the filters applied will affect the sort. You can
choose the motif sorting feature using the "Motifs:" menu.

If CentriMo is searching for locally enriched regions (not just centrally
enriched regions), then multiple regions may be found per motif, and
the "Regions:" menu will also be displayed. In this case,
CentriMo first sorts all regions using the feature
shown in the "Regions:" menu, and then it sorts the highest-ranked
region of each motif according to the feature shown in the "Motifs:" menu.

Unless you check the box next to the "Regions:" menu, it will automatically
show the same feature as the "Motifs:"
menu (or "*E*-value" if a motif-only feature is chosen in the "Motifs:" menu).

**Note:**The motif *p*-value shown in the plot legend will always be for
the region with the lowest *p*-value, and therefore may not match the value
shown in the table "*p*-value" column
when the "Regions:" menu is not set to "*p*-value".

[
close ]

The name of the database (file name) that contains the motif.

[
close ]

A name for the motif that is unique in the motif database file.

[
close ]

An alternate name of the motif that may be provided in the motif database file.

[
close ]

The expected number motifs that would have least one
region as **comparatively** enriched for
best matches to the motif as the reported region in the
**positive** sequences compared with the **negative**
sequences.

The Fisher *E*-value is the (one-sided) *p*-value of
the Fisher exact test that **at least** as many best matches
in the region in the positive sequences that contain at least
one match, multiplied by the number of motifs in the input database(s).
The Fisher exact test *p*-value is corrected for the number
of regions and score thresholds tested ("Multiple Tests").

The Fisher exact test assumes that the probability that the best match
(if any) falls into a given region is the same for all
positive and negative sequences.

[
close ]

The probability that any tested region would be as enriched for
best matches to this motif as the reported region is.

By default the *p*-value is calculated by using the one-tailed
binomial test on the number of sequences with a match to the
motif ("Total Matches") that have their best match in the reported region ("Region Matches"),
corrected for the number of regions and score thresholds tested ("Multiple Tests").
The test assumes that the probability that the best match in a sequence falls
in the region is the region width divided by the number of
places a motif can align in the sequence (sequence length minus motif
width plus 1).

When CentriMo is run in discriminative mode with a negative set of sequences, the
*p*-value of a region is calculated using the Fisher exact test
on the enrichment of best matches in the positive sequences ("Region Matches")
relative to the negative sequences ("Negative Region Matches"),
corrected for the number of regions and score thresholds tested ("Multiple Tests").
The test assumes that the probability that the best match
(if any) falls into a given region is the same for all
positive and negative sequences.

[
close ]

The expected number motifs that would have least one region as enriched for
best matches to the motif as the reported region.
The *E*-value is the *p*-value
multiplied by the number of motifs in the input database(s).

[
close ]

The Matthew's Correlation Coefficient (MCC) gives a measure of the ability
of the motif to discriminate the positive sequences from the negative sequences:

MCC = [TP\*TN - FP\*FN] / [(TP + FP) \* (TP + FN) \* (TN + FP) \* (TN + FN)]

where

:   TP is the number of positive sequences with a best match in the reported region,
:   FP is the number of negative sequences with a best match in the reported region,
:   TN is the number of negative sequences without a best match in the reported region, and
:   FN is the number of positive sequences without a best match in the reported region.

MCC ranges from -1 to +1, where a +1 result indicates that the occurrence
of the best match to the motif in the reported region perfectly discriminates positive
sequences from negative sequences.

[
close ]

This is the score threshold (in bits) for determining if a sequence contains a match to this motif.

When score optimization is enabled, this column lists the score
threshold that gives the best *p*-value. Otherwise it
shows the smallest score found that was above the minimum score threshold
specified to CentriMo.

[
close ]

The width (in sequence positions) of the most enriched region.
A best match to the motif is counted as being in the region if
the center of the motif falls in the region.

[
close ]

The number of (positive) sequences whose **best** match to the motif
falls in the reported region.

**Note:** This number may be less than the number of (positive) sequences that
have a best match in the region. The reason for this is that a sequence may
have many matches that score equally best. If *n* matches have the
best score in a sequence, 1/*n* is added to the appropriate bin
for each match.

[
close ]

The number of **negative** sequences where the **best** match to
the motif falls in the reported region. This value is rounded but the
underlying value may contain fractional counts.

**Note:** This number may be less than the number of **negative**
have a best match in the region. The reason for this is that a sequence may
have many matches that score equally best. If *n* matches have the
best score in a sequence, 1/*n* is added to the appropriate bin
for each match.

[
close ]

The number of sequences containing a match to the motif above the
score threshold ("Score Threshold").

[
close ]

The number of **negative** sequences containing a match to the motif
above the minimum score threshold. When score optimization is enabled the
score threshold may be raised higher than the minimum.

[
close ]

The probability that any tested region in the **negative**
sequences would be as enriched for best matches to this motif according
to the Binomial test.

Use the filter to display only motifs differentially enriched in both
datasets (low *p*-value and high negative *p*-value).

[
close ]

The maximum probability that the best match occurs at any single sequence position.
If the smoothing window size ("Window:", to right of graph) is set to "1", then this is value is
the maximum value of the match-probability curve.

[
close ]

This is the number of multiple tests (*n*) done for this motif. It was
used to correct the original *p*-value of a region for multiple tests
using the formula

:   p' = 1 - (1-p)^n

where *p* is the uncorrected *p*-value.
The number of multiple tests is the number of regions considered times the
number of score thresholds considered. It depends on the motif length, sequence length,
and the type of optimizations being done (central enrichment, local enrichement, score
optimization).

[
close ]

Location of the center of the most enriched region.

[
close ]

The text box lists the sequence identifiers for sequences which have at
least one of their best matches in the most significant region of **all** the
selected motifs.

The "Intersection" subheading gives the number of identifiers in the
text box and their percentage out of the total number of input sequences.

The "Union" subheading lists the number and percentage of
sequences that have at least one of their best matches in the most
significant region of **any** of the selected motifs and their
percentage out of the total number of input sequences.

Note that the number of sequences with a match to a given motif in
its best region may be larger than the value of "Region Matches". This is because
a sequence may have multiple equally best matches and in that case a
fractional match count is assigned to each of them when "Region Matches" is computed.

[
close ]

When more than one significant, non-overlapping region is found,
they can be shown (and hidden again) by clicking the arrow.

By default the regions are sorted by *E*-value, but this can be
changed by the menu on the right of the page.

[
close ]

Sequence position where the (unsmoothed) match-probability curve for this motif
attains its maximum. Set the smoothing window size ("Window:", to right of graph) to
"1" to see the unsmoothed match probability curve.

[
close ]

For further information on how to interpret these results or to get a
copy of the MEME software please access
http://meme.nbcr.net.

If you use CentriMo in your research, please cite the following paper:

Motif Probability Graph
  |  
Enriched Motifs
  |  
Input Files
  |  
Program information


# Javascript is required to view these results!

# Your browser does not support canvas!

## Results

#### Motif Probability Graph (score ≥ )

#### Options

##### Plotting

##### Unused Colours

##### Graph

Smoothing:

Moving Average
Weighted Moving Average

Window:

Legend:

Disabled
Enabled (click on graph to move)

Negative sequences:

Not plotted
Plotted as dashed line

#### Enriched motifs (*E*-value ≤ using the test)

| ☒ | Database | ID | Name | *E*-value | Fisher *E*-value | *p*-value | Negative *p*-value | MCC | Region Center | Region Width | Region Matches | Total Matches | Negative Region Matches | Negative Total Matches | Max Probability | Max Probability Location | Multiple Tests | Score Threshold | Other Regions |
| --- | --- | --- | --- | --- | --- | --- | --- | --- | --- | --- | --- | --- | --- | --- | --- | --- | --- | --- | --- |

#### Matching sequences (out of )

##### Union: 0 sequences (0%).

##### Intersection: 0 sequences (0%).

#### Filter & Sort

##### Filters

Top

Database is

ID matches

Name matches

*E*-value ≤

Fisher *E*-value≤

Region Width ≤

Negative set *E*-value ≥

##### Sort

Motifs:

Regions:

#### Columns to display

Show Database

Show ID

Show Name

Show *E*-value

Show *p*-value

Show Fisher *E*-value

Show Matthew's Correlation Coefficient

Show Region Center

Show Region Width

Show Region Matches

Show Total Matches

Show Negative *p*-value

Show Negative Region Matches

Show Negative Total Matches

Show Max Probability

Show Max Probability Location

Show Multiple Tests

Show Score Threshold

## Input Files

#### Sequences

| Database | Source | Sequence Count |
| --- | --- | --- |
|  |  |  |

#### Negative sequences

| Database | Source | Sequence Count |
| --- | --- | --- |
|  |  |  |

#### Motifs

| Database | Source | Motif Count |
| --- | --- | --- |

##### CentriMo version

(Release date: )

##### Reference

Timothy L. Bailey and Philip Machanick,
"Inferring direct DNA binding from ChIP-seq", *Nucleic Acids Research*, **40**:e128, 2012.

##### Command line summary
